# Bone Regeneration of Peri-Implant Defects Using a Collagen Membrane as a Carrier for Recombinant Human Bone Morphogenetic Protein-2

**DOI:** 10.1155/2018/5437361

**Published:** 2018-06-25

**Authors:** Yoo-Kyung Sun, Jae-Kook Cha, Daniel Stefan Thoma, So-Ra Yoon, Jung-Seok Lee, Seong-Ho Choi, Ui-Won Jung

**Affiliations:** ^1^Department of Periodontology, Research Institute for Periodontal Regeneration, College of Dentistry, Yonsei University, Seoul, Republic of Korea; ^2^Clinic for Fixed and Removable Prosthodontics and Dental Material Science, University of Zurich, Zurich, Switzerland

## Abstract

This study is designed to determine the effect of collagen membrane (CM) soaked with bone morphogenetic protein-2 (rhBMP-2) for the treatment of peri-implant dehiscence defects.* Material and Methods*. Three treatment groups were allocated at each defect in 5 dogs: (i) collagenated synthetic bone (OC) and CM soaked with rhBMP-2 (BMP group), (ii) OC and CM soaked with saline (nonBMP group), and (iii) no further treatment (control group). Titanium pins were used to stabilize the membranes in two dogs. Radiographic and histomorphometric analyses were performed 4 weeks later.* Results*. The median augmented volumes were 4.27 mm^3^, 6.24 mm^3^, and 2.75 mm^3^ in the BMP, nonBMP, and control groups, respectively; the corresponding median first bone-to-implant contact (fBIC) distances were 3.25 mm, 3.08 mm, and 2.56 mm (*P* > 0.05). The placement of pins (with the BMP and nonBMP groups pooled) significantly improved bone regeneration: the augmented volumes were 17.60 mm^3^ with pins and 3.68 mm^3^ without pins (*P* = 0.024), with corresponding fBIC distances of 2.25 mm and 3.31 mm, respectively (*P* < 0.001).* Conclusions*. The addition of rhBMP-2 to CM failed to improve bone regeneration of peri-implant dehiscence defects compared to using an unsoaked CM after 4 weeks. However, the stabilization of CMs using pins positively influenced the outcomes.

## 1. Introduction

Guided bone regeneration (GBR) using collagen membrane (CM) is a well-documented treatment modality for augmenting localized peri-implant bone defects, with many clinical and preclinical studies demonstrating that exposed implant surfaces can be successfully augmented [1–5]. However, this type of resorbable membrane appears to result in insufficient space maintenance, which is reportedly due to the pressure from the covering flap resulting in membrane collapse [3, 6]. Recent research has focused on techniques and materials to overcome these drawbacks [7–9].

Recombinant human bone morphogenetic protein-2 (rhBMP-2) has well-documented osteogenic properties that significantly improve bone regeneration [10–13]. Recent reviews have also considered rhBMP-2 to be the most promising bioactive molecules for bone regeneration [14–16].

Clinical considerations mean that dental implants need to be placed in prosthetically ideal positions, which often results in buccal dehiscence defects. Several preclinical and clinical studies have evaluated rhBMP-2 in combination with various bone-substitute materials for localized bone regeneration for this type of peri-implant defect [10, 11, 17, 18]. RhBMP-2 was combined with the bone-substitute material in all of these studies; although this resulted in successful treatment outcomes and superiority compared to control groups, the outcomes were to some extent controversial and limited by the clinical applicability of soaking bone-substitute materials with rhBMP-2. One option would be to use a CM as a carrier for rhBMP-2, since this would potentially be advantageous in being closer to the highly osteogenic periosteum containing abundant mesenchymal cells. In addition, a rapid bone formation on the outer side of the defect might result in a more stable augmented area. Based on this assumption, Chang and colleagues used a CM as a carrier for rhBMP-2 for primary horizontal bone augmentation, demonstrating a proof of concept [18]. However, this combination has not previously been evaluated for the clinically more common peri-implant defects.

Therefore, the aim of the present study was to determine the effect of a CM soaked with rhBMP-2 for the treatment of peri-implant dehiscence defects.

## 2. Material and Methods

### 2.1. Study Design

The present experiments were designed as a controlled preclinical study involving five mongrel dogs. The dogs were aged 12–15 months and a mean body weight of 30 kg. They had no systemic disease and showed a healthy periodontium and intact dentition. The study was performed in accordance with the Animal Care and Use Committee, Yonsei Medical Center, Seoul, Korea (permission no. 2011-0188).

### 2.2. Experimental Materials

The following materials were used in the study:

1. Titanium implants with a sandblasted and acid-etched surface (3.8 mm in diameter and 8 mm long) (Implantium®, Dentium, Seoul, Korea).
2. A collagenated synthetic bone (OC; OSTEON™ Collagen, Dentium) consisting of a particulate bone-substitute material [70% hydroxyapatite (HA) and 30%  *β*-tricalcium phosphate] and a type I collagen.
3. A resorbable CM containing HA particles (HA collagen membrane, GENOSS, Suwon, Korea).
4. rhBMP-2 (Cowellmedi, Busan, Korea) at a concentration of 0.5 mg/ml, which was obtained by reconstituting and diluting rhBMP-2 in a buffer solution. The HA-containing CMs were soaked in 0.2 ml of rhBMP-2 solution for 15 minutes at room temperature.

### 2.3. Surgical Procedures

Oral prophylaxis was applied to all dogs prior to the surgical intervention. Details of the surgical procedures are available elsewhere [19]. In brief, general anesthesia as well as local infiltration anesthesia at the surgical sites was applied. Crevicular incisions were then made from the second premolar to the first molar, and two vertical incisions were made on the buccal gingiva. Following hemisectioning, the second, third, and fourth premolars and the first molar were extracted on one side of the mandible. The buccal bone plates were removed, resulting in an acute defect with dimensions of 5 mm (apico-coronal width) by 5 mm (bucco-oral depth) and extending from the second to the fourth premolar. Primary wound closure was then performed. The sutures were removed 10 days later.

### 2.4. Implant Placement and Guided Bone Regeneration

**Figure 1:**
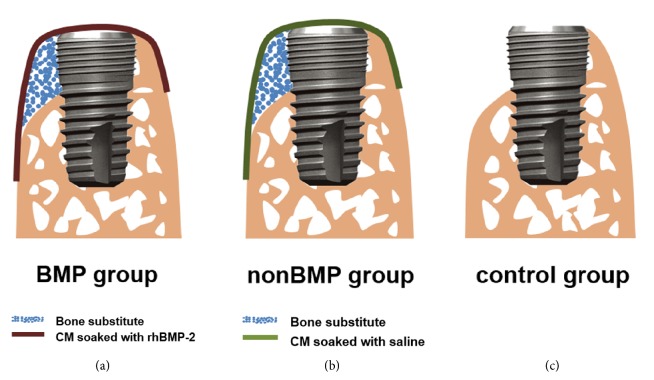
Schematic drawings of the surgery design: (a) BMP group, (b) nonBMP group, and (c) control group. Blue circles, bone-substitute materials; dark-red line, CM soaked with rhBMP-2; green line, CM soaked with saline; BMP group, cylinder-type bone-substitute material covered by CM soaked with rhBMP-2; nonBMP group, cylinder-type bone-substitute material covered by CM soaked with saline; control group, no further treatment.

**Figure 2:**
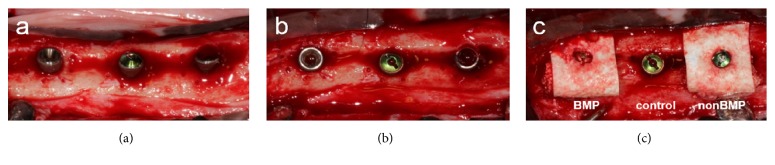
Clinical photographs of the bone augmentation procedure applied to peri-implant dehiscence defects: (a) buccal and (b) occlusal views after implant placement and (c) GBR treatment performed on dehiscence defects according to group assignment. The control group was placed at the center implant site, and the BMP and nonBMP groups were randomly allocated to the mesial and distal implant sites.

Twelve weeks later, dental implants were placed and simultaneous GBR was performed (Figure 1). Following flap reflection, the healed ridge was flattened and three implants were placed with their platforms flush with the lingual bone crest. This resulted in a peri-implant dehiscence defect with a height of 3 mm at the buccal aspect (Figure 2(a)). The cortical bone plate was perforated in the vicinity of the implants, and GBR was performed. The following three treatment modalities were applied (Figure 2(c)):

1. OC and a CM containing HA particles soaked with rhBMP-2 (BMP group).
2. OC and a CM containing HA particles soaked with sterile saline (nonBMP group).
3. No GBR (control group).

The control group was always located at the center implant site to minimize the influence of the BMP molecule on adjacent groups, while the BMP and nonBMP groups were allocated to the mesial or distal implant sites randomly.

In group BMP, the OC was grafted on the peri-implant defect and covered with a CM containing HA particles soaked with rhBMP-2. In group nonBMP, the OC was grafted on the peri-implant defect and then covered with a CM containing HA particles soaked with saline. No overaugmentation was attempted in either of these groups. In two of the five dogs, two titanium pins (Frios® membrane tacks, DENTSPLY Implants, Mannheim, Germany) were used to stabilize the membranes in the BMP and nonBMP groups, while no fixation pins were applied in the remaining three dogs. In addition, the CM was perforated on top of the implant and immobilized by a cover screw. No graft material or membrane was used on the control group.

Periosteal releasing incisions were subsequently made and primary wound closure was achieved using a resorbable suture material (Monosyn® 4.0 Glyconate Monofilament, B. Braun, Tuttlingen, Germany). The sutures were removed 10 days later. The dogs were sacrificed by an overdose of sodium pentobarbital 4 weeks after implant placement and GBR surgery.

### 2.5. Radiographic Analysis

**Figure 3:**
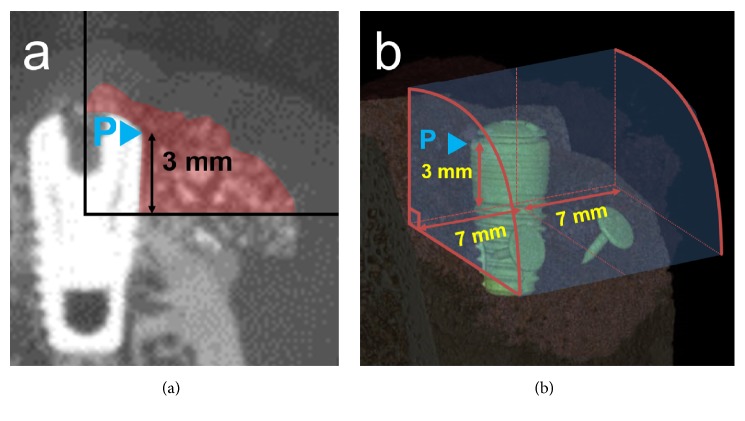
Schematic configuration of the TAV (mm^3^). (a) The lower border of the TAV was located 3 mm below the implant platform. The coronal border was defined by the most-coronal location of radiopaque tissue. The buccolingual extension of the TAV ranged from the center of the implant to the most-buccal radiopaque tissue (at an angle of 90° to the implant surface). (b) The mesiodistal borders of the TAV were confined by vertical lines 7 mm from the center of the implant surface. The implant itself was excluded from the TAV. P, implant platform.

The specimens were scanned using micro-CT (SkyScan 1072, SkyScan, Aartselaar, Belgium) and the total augmented volume (TAV, mm^3^) was measured, which represented the regenerated tissue surrounding the implant. Mineralized tissue was considered to be indicated in the images by grayscale values from 39 to 52 (defined as radiopaque tissue). The lower border of the TAV was located 3 mm below the implant platform, and the coronal border was defined by the most-coronal location of radiopaque tissue. The buccolingual extension of the TAV ranged from the center of the implant to the most-buccal radiopaque tissue (at an angle of 90° to the implant surface). The mesiodistal borders of the TAV were confined by vertical lines 7 mm from the center of the implant surface (Figure 3). The implant itself was excluded from the TAV.

**Figure 4:**
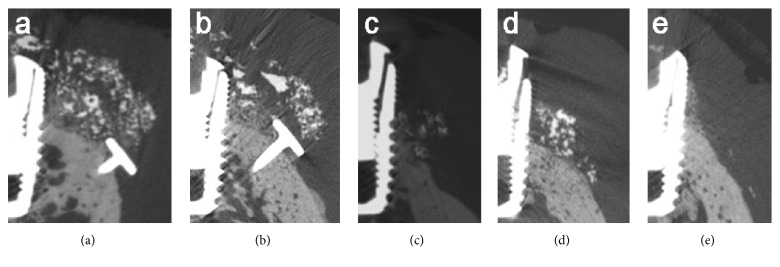
Cross-sectional radiographic images demonstrating variations both between and within the groups: (a) BMP group with pins, (b) nonBMP group with pins, (c) BMP group without pins, (d) nonBMP group without pins, and (e) control group. In the BMP and nonBMP groups, the shape of the augmented region appeared to be predominantly influenced by the use of pins.

**Figure 5:**
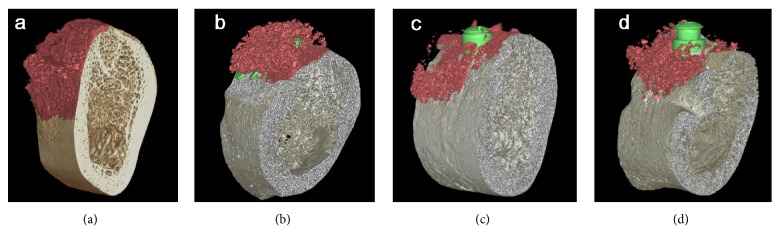
Three-dimensional reconstructed color-coded images of the total augmented area: (a) BMP group with pins, (b) nonBMP group with pins, (c) BMP group without pins, and (d) nonBMP group without pins. No remaining peri-implant defects were observed in the specimens with pins, in contrast with the sites without pins and control sites without GBR.

Cross-sectional images of each group are presented in Figure 4. The total augmented materials were painted using OnDemand 3D software (Cybermed, Seoul, Korea) to facilitate identification of the bone regeneration (Figure 5).

### 2.6. Histologic Analysis

Block specimens were harvested that included the implants and grafted sites with surrounding hard and soft tissues, and they were fixed in 10% neutral buffered formalin for 10 days. The specimens were then trimmed and dehydrated in ethanol before being embedded in methyl methacrylate. Specimens were cut in the center of the implant sites in a buccolingual plane and stained with hematoxylin-eosin and Masson's trichrome. The final thickness of the sections was 20 *μ*m.

### 2.7. Descriptive Histology

The histology sections were examined under a light microscope (BX-50, Olympus Optical, Tokyo, Japan) to identify relevant structures such as the implants, new bone formation, bone-substitute material, nonmineralized tissue, and the remaining peri-implant defect.

### 2.8. Histomorphometric Analysis

**Figure 6:**
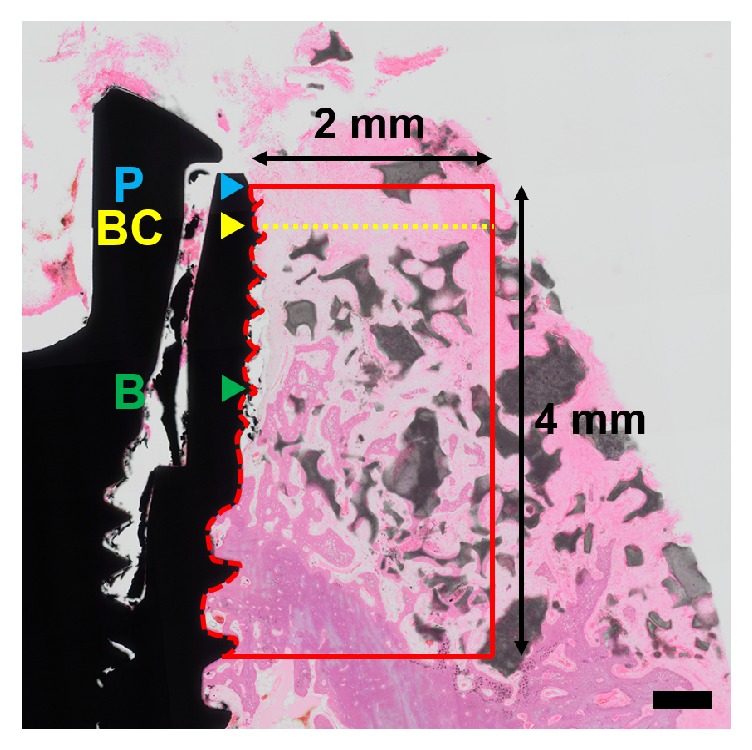
Schematic configuration for the histomorphometric analysis. P, implant platform; BC, most-coronal buccal bone; B, first bone-to-implant contact; red box, AOI. Scale bar = 500 *μ*m.

Histomorphometric measurements were made at the buccal aspect of all implants. An image-analysis program (Adobe Photoshop CS6, Adobe Systems, San Jose, CA, USA) was used to assess the following landmarks: implant platform (P), the first bone-to-implant contact (B), and the most-coronal buccal bone (BC) (Figure 6).

#### 2.8.1. Linear Measurements

The following linear measurements were made:

1. Distance between the implant platform (P) and the first bone-to-implant contact (B) (fBIC, mm).
2. Vertical distance between the most-coronal buccal bone (BC) and the implant platform (P) (P-BC, mm).
3. The bone-to-implant contact (BIC, %), measured along the implant surface between the implant platform (P) and extending 4 mm apically.

#### 2.8.2. Area/Surface Measurements

A rectangular area of interest (AOI) was defined on the buccal side of the implants and included the following dimensions: coronal (implant platform), apical (4 mm apically toward the implant platform), and horizontal (2 mm from the implant surface). The ratios of the following outcomes to the AOI area were measured:

1. Newly formed bone (NB, %).
2. Residual bone-substitute material (RBS, %).
3. Nonmineralized tissue (NMT, %).

### 2.9. Statistical Analysis

Mean and standard deviation values were calculated in each group. One-way analysis of variance and the Bonferroni post hoc test were used to assess the clinical benefit of soaking the CM with the rhBMP-2 solution. The independent *t*-test was used to identify differences among groups related to the use of pins. The cutoff for statistical significance was *P* < 0.05.

## 3. Results

### 3.1. Clinical Findings

All of the dogs remained healthy, with no wound dehiscence or membrane exposure being detected throughout the experiments. All of the obtained samples were included in the analyses.

### 3.2. Radiographic Analysis

The cross-sectional images demonstrated variations both between and within the groups (Figure 4). In the BMP and nonBMP groups, the shape of the augmented region appeared to be predominantly influenced by the use of pins. No remaining peri-implant defects were observed in the specimens with pins, in contrast with the sites without pins and control sites without GBR (Figure 5).

The median TAV values were 4.27 mm^3^, 6.24 mm^3^, and 2.75 mm^3^ in the BMP, nonBMP, and control groups, respectively (all *P* > 0.05) (Table 1). When the groups were divided according to the presence or absence of fixation pins, the median TAV was significantly higher in the group with pins (17.60 mm^3^) than in that without pins (3.68 mm^3^) (*P* = 0.024) (Table 2).

### 3.3. Histologic Observations and Histomorphometric Analysis

**Figure 7:**
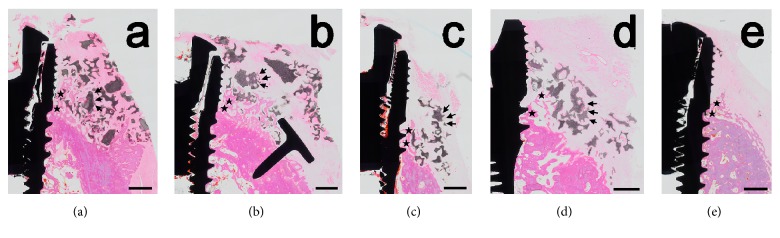
Histologic views presenting the results of GBR: (a) BMP group with pins, (b) nonBMP group with pins, (c) BMP group without pins, (d) nonBMP group without pins, and (e) control group. The results for defect resolution were not consistent, as demonstrated by some specimens in both the BMP and nonBMP groups exhibiting complete regeneration and others with remaining peri-implant bone defects. Complete resolution of the dehiscence defects was consistently observed in the histology specimens of the BMP and nonBMP groups using pins, while the positioning of bone-substitute particles was disrupted and bone regeneration did not occur uniformly in the group without pins. In the control sites, the size of the peri-implant defects appeared to be similar to the situation prior to augmentation. Asterisk, newly formed bone; arrow, residual bone-substitute material. Scale bar = 1 mm.

In the two GBR groups (i.e., BMP and nonBMP), bone formation was generally observed along the implant surfaces (Figure 7). However, defect resolution was not consistent, with some specimens in both groups exhibiting complete regeneration and others with remaining peri-implant bone defects. In control sites, the size of the peri-implant defects appeared to be similar to that prior to augmentation. Complete resolution of the dehiscence defects was consistently observed in the histology specimens of the BMP and nonBMP groups using pins, with new bone having formed at the apical border of the bone defect and around the bone-substitute particles. In contrast, the positioning of the bone-substitute particles was disrupted and bone regeneration did not occur uniformly in the group without pins.

The median fBIC distances were 3.25 mm, 3.08 mm, and 2.56 mm in the BMP, nonBMP, and control groups, respectively, with corresponding median BIC values of 11.90%, 18.24%, and 21.96%.

Within the AOI, the median NBs were 12.84%, 8.06%, and 21.75% in the BMP, nonBMP, and control groups, respectively. The median RBS was 1.31% in the BMP group and 12.43% in the nonBMP group. There were no significant intergroup differences (*P* > 0.05). All of the data are reported in Table 3.

All of the measured histomorphometric and radiographic values (except for RBS in histomorphometric analyses) differed significantly with the presence or absence of pins when 10 specimens were divided according to the use of pins (Table 2). The median NB was 37.03% with pins and 4.87% without pins in the BMP group and 47.18% with pins and 7.90% without pins in the nonBMP group. BIC was higher in both the BMP and nonBMP groups with pins (40.38% and 33.19%, respectively) than without pins (10.49% and 10.69%, respectively) (Table 3).

## 4. Discussion

This study has revealed that (i) the addition of rhBMP-2 to a CM does not significantly improve peri-implant bone regeneration and (ii) fixation of the CM using pins significantly increases bone regeneration compared to using a CM without pins.

This study was designed to evaluate the usefulness of a CM as a carrier for rhBMP-2 for guided bone regeneration at peri-implant dehiscence defects. Previous experiments that employed a similar protocol using animals and 30-mm peri-implant defects demonstrated that this model can serve as a valid alternative to clinical studies [20, 21].

Various preclinical models have demonstrated that rhBMP-2 improves bone regeneration by accelerating osteogenesis predominantly at the early stage of wound healing [22, 23]. In addition, the healing time is reportedly twofold shorter in dogs than in humans [24], and so earlier observation periods were established in this present study when evaluating the efficacy of rhBMP-2 compared to the previous experiments. In the present study, the bone formation at 4 weeks was greater in the GBR groups (by up to 47%) than at the untreated control sites (22%). This is in line with the reporting greater bone formation for GBR groups compared to the control sites, although those results were for longer healing periods of 8 and 16 weeks [21]. However, the addition of rhBMP-2 to a CM in the present study was not beneficial to bone regeneration compared to a CM soaked in saline at the early stage of healing of 4 weeks.

The other main difference between these two studies—apart from the healing period—was in the type of carrier material used. Various carrier materials have been used for rhBMP-2 [25–27]. It was speculated that using a CM as a carrier for rhBMP-2 could enhance the osteoinductivity of the periosteum due to the close proximity of the membrane and the periosteum. It was also speculated that an rhBMP-2 carrier in such a location would result in faster bone regeneration at the borders of the defect area, thereby ideally leading to shell-like bone formation, which could further compensate the disadvantages of a resorbable nonspace-maintaining membrane. However, this outcome was not observed in the present study, with no shell-like bone formation observed in any of the samples. In a preliminary study that used a GBR protocol and materials similar to those in the present study [18], histomorphometric analyses revealed that new bone formed closer to the membrane in the group with an rhBMP-2-loaded membrane than in the group with rhBMP-2 loaded on the bone-substitute material. This was observed as the new bone formed directly underneath the membrane in the group with rhBMP-2 loaded membrane. The differences in the observed bone regeneration pattern between these two studies might have been due to different healing periods (8 weeks versus 4 weeks), the placement of implants versus GBR alone, and the use of fixation pins.

Stability of the surgical site and space maintenance are considered to be essential for successful bone regeneration. The stabilization of a GBR site appears to be the critical factor governing the amount of bone formation. In a clinical study, it was found that bone formation was superior when using fixation pins [4]. This was subsequently supported by two in vitro studies that evaluated the effect of wound closure on the stability of GBR sites using various material combinations [6, 28], which found that the bone-substitute material moved apically upon wound closure. However, CMs were used at all sites, and it is well known that this type of membrane is weak mechanically and so may not be able to resist compressive forces. This can result in collapse of the membrane and the above-mentioned displacement of bone-substitute material. However, the studies have demonstrated that applying additional fixation pins can reduce the membrane displacement by 50% at the level of the implant shoulder. The clinical recommendation was to add fixation pins when CMs are used in combination with particulate graft materials [6]. Fixation pins were used in two of the five dogs in the present study. The failure to standardize the experimental method was due to not being possible to fix the pins on thick, rigid cortical bone without deformation. The results demonstrate the beneficial bone formation at sites where pins are used. However, it could be considered to use alternative fixation materials, such as miniscrews which have better strength than pins in case of performing GBR on a rigid bony plate.

Apart from the fixation pins, more-rigid barrier membranes [29] or more-stable grafting materials provide further advantages in stabilizing the augmented site [30]. This was implemented in the present study by combining a cylindrical type of synthetic bone-substitute material with a type I collagen matrix and HA-coated CMs. This bone-substitute material incorporating a collagen matrix was considered to resist compressive forces and to support the augmented ridge volume [31]. An in vitro study using a similar membrane coated with HA demonstrated a significantly enhanced chemical stability and an improved mechanical structure of the membrane [32]. Moreover, the cross-linked chemical structure stiffened the CM and thereby reduced the risk of collapse [33]. Such a membrane theoretically exhibits all the characteristics necessary to support space maintenance. However, in clinical experiments the increased stiffness resulted in major difficulties in handling the membrane and applying it properly to the defect site, which meant that displacement of the graft material could not be avoided.

The outcomes of this study are limited by several factors, including the relatively small number of experimental animals, the use of pins in only two of the five dogs, the handling difficulties with the membrane resulting in suboptimal clinical outcomes, and the short observation period. Further studies involving larger numbers of animals and appropriate statistical analyses are required to confirm the results of this study.

## 5. Conclusion

The use of rhBMP-2 soaked on a CM as a carrier material did not result in superior bone formation compared to control sites without rhBMP-2. However, the use of fixation pins to stabilize the CMs did exert a positive effect on peri-implant bone regeneration.

## Figures and Tables

**Table 1:** Measured the total augmented volume (TAV) values. *P* value for intergroup comparison = 0.376. *n* = number of sites/dogs.

Group	TAV (mm^3^)
BMP group	4.27 (3.08–18.23)
with pins (*n* = 2)	20.87 (19.55–22.20)
without pins (*n* = 3)	3.08 (1.68–3.68)
nonBMP group	6.24 (5.71–7.52)
with pins (*n* = 2)	12.25 (9.88–14.62)
without pins (*n* = 3)	5.71 (3.15–5.98)
Control group	2.75 (1.98–4.80)

Data are median (interquartile range) values.

**Table 2:** Radiographic and histomorphometric outcomes, with data for the BMP and nonBMP groups pooled according to the use of pins.

		With pins (*n* = 4)	Without pins (*n* = 6)	*P*
Radiographic volume	TAV, mm^3^	17.60 (7.52–23.52)	3.68 (0.27–5.71)	0.024

Linear measurements	BIC, %	40.38 (18.24–48.15)	10.59 (2.61–26.12)	0.027
	fBIC, mm	2.25 (1.95–2.38)	3.31 (3.08–0.87)	<0.001
	P-BC, mm	0.59 (0.00–1.27)	2.91 (2.51–3.44)	0.001

Measurements in the AOI	NB, %	42.54 (28.69–54.66)	6.56 (3.40–12.84)	0.006
	RBS, %	8.03 (0.00–19.01)	6.86 (0.00–23.66)	0.908
	NMT, %	53.46 (32.92–56.66)	84.94 (68.29–95.29)	0.002

Data are median (interquartile range) values.

*n* = number of sites/dogs. TAV (mm^3^) = the total augmented volume; BIC (%) = the bone-to-implant contact; fBIC (mm) = distance between the implant platform and the first bone-to-implant contact; P-BC (mm) = vertical distance between the most-coronal buccal bone and the implant platform; NB (%) = newly formed bone; RBS (%) = residual bone-substitute material; NMT (%) = nonmineralized tissue.

**Table 3:** Histomorphometric measurements.

	Linear measurements			Measurements in the AOI		
Group	BIC, %	fBIC, mm	P-BC, mm	NB, %	RBS, %	NMT, %
BMP group	11.90 (10.49–34.65)	3.25 (2.29–3.36)	2.64 (1.27–2.75)	12.84 (4.87–28.69)	1.31 (0.00–12.42)	82.71 (54.63–87.16)
with pins (*n* = 2)	40.38 (37.52–43.25)	2.25 (2.23–2.27)	0.64 (0.32–0.95)	37.03 (32.86–41.20)	9.51 (4.75–14.26)	53.46 (52.88–54.05)
without pins (*n* = 3)	10.49 (6.55–11.20)	3.36 (3.31–3.62)	2.75 (2.70–3.04)	4.87 (4.14–8.86)	1.31 (0.65–6.86)	87.16 (84.94–91.22)
nonBMP group	18.24 (10.69–26.12)	3.08 (2.38–3.17)	2.51 (0.65–3.06)	8.06 (7.90–39.71)	12.43 (3.63–19.56)	68.29 (56.66–75.23)
with pins (*n* = 2)	33.19 (25.72–40.67)	2.17 (2.06–2.27)	0.59 (0.56–0.62)	47.18 (43.45–50.92)	8.03 (5.83–10.23)	44.79 (38.85–50.73)
without pins (*n* = 3)	10.69 (10.33–18.41)	3.17 (3.13–3.31)	3.06 (2.79–3.25)	7.90 (6.56–7.98)	19.56 (9.78–21.61)	75.2 (71.76–83.66)
Control group	21.96 (8.16–5.04)	2.56 (2.55–3.74)	2.04 (1.60–3.56)	21.75 (3.19–22.91)	0.00	78.25 (77.09–96.81)
*P*	0.873	0.664	0.679	0.550	0.313	0.152

Data are median (interquartile range) values.

*n* = number of sites/dogs.

None of the outcome measured differed significantly between the three groups (*P* > 0.05).

## Data Availability

The data used to support the findings of this study are available from the corresponding author upon request.
